# Supplementary material for: Characterization and Genome Study of Novel Lytic Bacteriophages against Prevailing Saprophytic Bacterial Microflora of Minimally Processed Plant-Based Food Products
Source: Int J Mol Sci. 2021 Nov 18;22(22):12460. doi: 10.3390/ijms222212460 (PMC8624825; doi:10.3390/ijms222212460)
Supplement: Supplementary file 1 [file ijms-22-12460-s001.zip › Table_S2.pdf]

Table S2. BLASTn alignment of homologs phages sequences (at the GenBank database) with isolated phages

Phage (complete genome)	Phage Family	Genome	Isolated Country	Isolation Source	Lab Host	Accession No.	Lenght (bp)	E value	Query coverage	Percent identity
***Enterobacter* phage KKP 3263**										
Escherichia phage vB_EcoP_SP7	*Autographiviridae*	Linear DNA	Portugal	Waste water from pig farming	No data	MT682707.1	39305	0.0	87%	92.52%
Escherichia phage Peacock	*Autographiviridae*	Linear DNA	USA	Waste water treatment plant influent sample	*Escherichia coli*	MK903279.1	39233	0.0	85%	93.76%
Salmonella phage vB Seyj3-1	*Autographiviridae*	Linear DNA	China	No data	*Salmonella* Typhi CMCC50071	MW416012.1	39619	0.0	85%	91.58%
Hafnia phage vB_HpaA_yong1	*Autographiviridae*	Linear DNA	China	River water	*Hafnia paralvei* strain LY-23	MK610268.1	40286	0.0	85%	92.16%
Escherichia phage vB_EcoP_GA2A	*Autographiviridae*	Linear DNA	Canada	Speed River	*Escherichia coli* O:45 strain C1328	KT990215.1	40470	0.0	85%	92.43%
Escherichia phage CLB_P1	*Autographiviridae*	Linear DNA	France	No data	No data	KC109329.1	40218	0.0	84%	93.81%
Escherichia phage PE3-1	*Autographiviridae*	Linear DNA	China	No data	*Escherichia coli* O153	KJ748011.1	39093	0.0	84%	92.70%
Escherichia phage JSS1	*Autographiviridae*	Linear DNA	China	No data	*Escherichia coli* O153	KX689784.2	39024	0.0	84%	92.66%

c.d. **Table S2.** BLASTn alignment of homologs phages sequences (at the GenBank database) with isolated phages

Phage (complete genome)	Phage Family	Genome	Isolated Country	Isolation Source	Lab Host	Accession No.	Lenght (bp)	E value	Query coverage	Percent identity
Escherichia phage LM33_P1	*Autographiviridae*	Linear DNA	France	No data	*Escherichia coli* LM33	LT594300.1	38979	0.0	84%	93.36%
Escherichia phage Mt1B1_P3	*Autographiviridae*	Linear DNA	France	Sewage water	*Escherichia coli* Mt1B1	MT496969.1	40313	0.0	84%	93.05%
Escherichia virus Vec13	*Autographiviridae*	Linear DNA	Russian Federation	Farm sewage	*Escherichia coli*	NC_048023.1	39747	0.0	84%	93.13%
Escherichia phage DY1	*Autographiviridae*	Linear DNA	China	Pearl River	*Escherichia coli*	MT808983.1	39817	0.0	84%	92.85%
Escherichia phage vB_EcoP_F	*Autographiviridae*	Linear DNA	United Kingdom	No data	*Escherichia coli* O18ac:K1:H7 ColV+	NC_047808.1	39300	0.0	84%	94.05%
Escherichia phage ST31	*Autographiviridae*	Circular DNA	USA	No data	*Escherichia coli* ST130	NC_047829.1	39693	0.0	84%	93.52%
Citrobacter phage SH3	*Autographiviridae*	Linear DNA	Canada	No data	*Citrobacter freundii* CF3	KU687349.1	39444	0.0	84%	94.29%
Enterobacteria phage EcoDS1	*Autographiviridae*	Linear DNA	USA	No data	*Escherichia coli*	EU734172.1	39252	0.0	84%	93.79%
***Serratia* phage KKP 3264**										
Escherichia phage arall	*Myoviridae*	Linear DNA	Denmark	Waste water	*Escherichia coli* K-12 MG1655	MN850584.1	145715	0.0	95%	98.02%
Escherichia phage vB_vPM_PD06	*Myoviridae*	Circular DNA	USA	No data	*Escherichia coli*	NC_052653.1	149506	0.0	95%	98.75%
Escherichia phage alia	*Myoviridae*	Linear DNA	Denmark	Waste water	*Escherichia coli* K-12 MG1655	NC_052655.1	147009	0.0	95%	98.03%

c.d. **Table S2.** BLASTn alignment of homologs phages sequences (at the GenBank database) with isolated phages

Phage (complete genome)	Phage Family	Genome	Isolated Country	Isolation Source	Lab Host	Accession No.	Lenght (bp)	E value	Query coverage	Percent identity
Escherichia phage vB_vPM_PD114	*Myoviridae*	Circular DNA	China	No data	*Escherichia coli*	MH675927.1	150354	0.0	95%	98.50%
Enterobacteria phage ECGD1	*Myoviridae*	Linear DNA	China	No data	No data	KU522583.1	146647	0.0	94%	98.73%
Escherichia phage vB_EcoM_PHB05	*Myoviridae*	Linear DNA	China	Waste water	Shiga toxin-producing *Escherichia coli*	NC_052652.1	147659	0.0	94%	99.03%
Escherichia phage inny	*Myoviridae*	Linear DNA	Denmark	Waste water	*Escherichia coli* K-12 MG1655	MN850601.1	147483	0.0	94%	97.59%
Enterobacteria phage phi92	*Myoviridae*	Linear DNA	Germany	No data	*Escherichia coli* K92	FR775895.2	148612	0.0	94%	97.96%
Escherichia phage outra	*Myoviridae*	Linear DNA	Denmark	Waste water	*Escherichia coli* K-12 MG1655	MN850645.1	145482	0.0	94%	98.54%
Escherichia phage muut	*Myoviridae*	Linear DNA	Denmark	Waste water	*Escherichia coli* K-12 MG1655	NC_052657.1	146307	0.0	94%	98.41%
***Enterobacter* phage KKP 3262**										
Klebsiella phage vB_KaeM_KaAlpha	*Myoviridae*	Linear DNA	USA	Raw sewage	*Klebsiella aerogenes*	MN013084.1	172334	0.0	96%	96.83%
Enterobacter phage PG7	*Myoviridae*	Linear DNA	China	Fishpond water	*Enterobacter cloacae* clinial isolate EC7	KJ101592.1	173276	0.0	95%	95.32%

c.d. **Table S2.** BLASTn alignment of homologs phages sequences (at the GenBank database) with isolated phages

Phage (complete genome)	Phage Family	Genome	Isolated Country	Isolation Source	Lab Host	Accession No.	Lenght (bp)	E value	Query coverage	Percent identity
Cronobacter phage Pet-CM3-4	*Myoviridae*	Linear DNA	Slovakia	Waste water	*Cronobacter malonaticus* LMG 23826	NC_055726.1	171975	0.0	94%	98.29%
***Citrobacter* phage KKP 3664**										
Citrobacter phage vB_CfrM_CfP1	*Myoviridae*	Linear DNA	Portugal	Waste water plant raw effluent	No data	KX245890.1	180219	0.0	96%	90.00%
Citrobacter phage Miller	*Myoviridae*	Linear DNA	USA	No data	No data	KM236237.1	178171	0.0	96%	94.83%
Buttiauxella phage vB_ButM_GuL6	*Myoviridae*	Linear DNA	Lithuania	No data	*Buttiauxella* sp. environmental isolate G6	MT334653.1	178039	0.0	96%	90.98%
Citrobacter phage vB_Cfr_Xman	*Myoviridae*	Linear DNA	USA	No data	*Citrobacter* sp.	MW021749.1	178331	0.0	95%	90.05%
Enterobacteria phage RB43, isolate RB43-GVA	*Myoviridae*	Linear DNA	France	No data	No data	HE858210.2	179836	0.0	95%	94.70%
Enterobacteria phage RB43, isolate RB43-GVA orf057::gfp	*Myoviridae*	Linear DNA	France	No data	No data	HE981739.1	179839	0.0	95%	94.70%
Enterobacteria phage RB43	*Myoviridae*	Linear DNA	USA	No data	No data	AY967407.1	180500	0.0	95%	94.69%
Escherichia phage Lw1	*Myoviridae*	Linear DNA	Ukraine	No data	*Escherichia coli* BL21 (DE3)	KC801932.2	176227	0.0	94%	90.91%
Citrobacter phage IME-CF2	*Myoviridae*	Linear DNA	China	Hospital sewage	*Citrobacter freundii*	KR869820.1	177688	0.0	94%	91.83%
Escherichia phage RB16	*Myoviridae*	Linear DNA	USA	No data	*Escherichia coli*	HM134276.1	176788	0.0	94%	95.05%
